# Comparison of two propensity score-based methods for balancing covariates: the overlap weighting and fine stratification methods in real-world claims data

**DOI:** 10.1186/s12874-024-02228-z

**Published:** 2024-06-03

**Authors:** Wen Wan, Manoradhan Murugesan, Robert S. Nocon, Joshua Bolton, R. Tamara Konetzka, Marshall H. Chin, Elbert S. Huang

**Affiliations:** 1 Section of General Internal Medicine, Department of Medicine, The University of Chicago, 5841 S. Maryland Ave, Chicago, MC, IL 2007, 60637 USA; 2 Department of Public Health Sciences, Department of Medicine, The University of Chicago, Chicago, IL USA; 3 Health Systems Science, Kaiser Permanente Bernard J. Tyson School of Medicine, Pasadena, CA USA; 4 Department of Information Systems, University of Maryland, Baltimore, MD USA

**Keywords:** Overlap weighting method (OW), Fine stratification method (FS), Covariate balance, Plasmode simulation method, Propensity score (PS), Average treatment effect (ATE)

## Abstract

**Background:**

Two propensity score (PS) based balancing covariate methods, the overlap weighting method (OW) and the fine stratification method (FS), produce superb covariate balance. OW has been compared with various weighting methods while FS has been compared with the traditional stratification method and various matching methods. However, no study has yet compared OW and FS. In addition, OW has not yet been evaluated in large claims data with low prevalence exposure and with low frequency outcomes, a context in which optimal use of balancing methods is critical. In the study, we aimed to compare OW and FS using real-world data and simulations with low prevalence exposure and with low frequency outcomes.

**Methods:**

We used the Texas State Medicaid claims data on adult beneficiaries with diabetes in 2012 as an empirical example (*N* = 42,628). Based on its real-world research question, we estimated an average treatment effect of health center vs. non-health center attendance in the total population. We also performed simulations to evaluate their relative performance. To preserve associations between covariates, we used the plasmode approach to simulate outcomes and/or exposures with *N* = 4,000. We simulated both homogeneous and heterogeneous treatment effects with various outcome risks (1-30% or observed: 27.75%) and/or exposure prevalence (2.5-30% or observed:10.55%). We used a weighted generalized linear model to estimate the exposure effect and the cluster-robust standard error (SE) method to estimate its SE.

**Results:**

In the empirical example, we found that OW had smaller standardized mean differences in all covariates (range: OW: 0.0–0.02 vs. FS: 0.22–3.26) and Mahalanobis balance distance (MB) (< 0.001 vs. > 0.049) than FS. In simulations, OW also achieved smaller MB (homogeneity: <0.04 vs. > 0.04; heterogeneity: 0.0-0.11 vs. 0.07–0.29), relative bias (homogeneity: 4.04–56.20 vs. 20–61.63; heterogeneity: 7.85–57.6 vs. 15.0-60.4), square root of mean squared error (homogeneity: 0.332–1.308 vs. 0.385–1.365; heterogeneity: 0.263-0.526 vs 0.313-0.620), and coverage probability (homogeneity: 0.0–80.4% vs. 0.0-69.8%; heterogeneity: 0.0-97.6% vs. 0.0-92.8%), than FS, in most cases.

**Conclusions:**

These findings suggest that OW can yield nearly perfect covariate balance and therefore enhance the accuracy of average treatment effect estimation in the total population.

**Supplementary Information:**

The online version contains supplementary material available at 10.1186/s12874-024-02228-z.

## Background

Due to infeasibility of running a randomized experiment, observational data are often used to estimate the population health effects of interventions. When estimating plausibly causal effects using observational data, it is necessary to reduce imbalance in the empirical distribution of the pretreatment confounders between the treated and control groups [1]. Lowering imbalance can reduce the degree of model dependence for the statistical estimation of causal effects [1–4], and thus reduces inefficiency and bias [1]. To achieve balanced covariates, propensity scores (PS) have become a cornerstone in observational studies aimed at estimating causal effects [5, 6]. PS are defined as the predicted probability of receiving a particular treatment (or exposure) for the given covariate realizations of a study subject.

In this paper, we study PS-based approaches to estimate the average treatment effect in the total population (ATE). There are three common types of balancing methods via PS: matching, stratifying, and weighting. Among matching methods, the PS matching method (PSM) is the most commonly used in practice [1]. It is simple and intuitive by reducing the multidimensional covariate space to one dimension. Despite its widespread adoption, a large sample size is required as it discards some subjects who are not matched. In addition, PSM has been shown to increase model “imbalance, inefficiency, model dependence, and bias,” which is not the case with most other matching methods [1]. Among the stratification methods, the most common one is to stratify subjects into five quintiles of PS. With the stratum boundaries determined by PS distribution in the exposed and the comparison group combined, it eliminates approximately 90% of bias due to measured confounding [7]. However, when exposure is infrequent, it may result in all exposed subjects being aggregated in one or more extreme strata [5, 8]. The fine stratification weights method (FS), a recent method, can solve this issue by increasing number of strata and by determining stratum boundaries based on PS distribution in exposed group only. It has been shown to gain greater efficiency than the traditional one [8]. Among the weighting methods, inverse probability weighting (IPW) is popular but performs poorly when some subjects have extreme PS [9–11]. The PS based overlap weighting method (OW), another recent method, overcomes IPW’s extreme weight issue and produces impressive covariate balance [12, 13]. 

OW has been theoretically proven to have small-sample exact balance property [12]. That is, it leads to exact balance on the mean of every covariate when the PS is estimated by a logistic regression. It is less sensitive to model misspecification compared to the inverse probability weighting method (IPW) in a simulation study [14]. Despite these features, to our knowledge, OW has only been evaluated by comparing with weighting methods such as IPW and trimmed IPW [9–14]. Little is known about the relative performance of OW compared with other types of balancing methods including matching and stratification methods [15]. In addition, OW has not been evaluated in large claims data with low prevalence exposure and/or with low frequency events (i.e., outcomes), a context in which optimal use of balancing methods is critical.

Furthermore, matching on PS is limited by exclusion of subjects without a suitable match leading to a non-representative population and a loss of statistical power [16]. PSM including 1:1, 1:5, and full matching have less model precision than FS in at least two claims studies [8, 17]. Therefore, we aimed to compare OW with FS only, both relatively new and promising methods, using real-world and simulated claims data in settings with infrequent exposure and/or with low prevalence outcomes.

## Methods

### Empirical example

We used a cohort of 42,628 Texas State Medicaid beneficiaries, aged 18–64, diagnosed with type 2 diabetes, who had at least one primary care visit between January 2012 and December 2012. About 10.55% (*n* = 4,498) of the patients received the majority of their primary care at federally qualified health centers (FQHCs) (exposure), while the rest (89.45%, *n* = 38,130) received care at non-FQHCs (control). Researchers analyzed whether or not those patients who had routine primary care at FQHCs had fewer hospitalizations and emergency room visits than the non-FQHC patients. Five continuous and 12 binary covariates were selected based on clinical relevance and previous literature. The empirical example has 10.55% exposure rate which is near rare (typically < 10% considered as rare) and hospitalization quite often is a rare outcome.

The study was reviewed by the University of Chicago Institutional Review Board and determined to be non-human subject research.

### Overlap weighting method (OW)

The OW method mimics a randomized trial by assigning appropriate weights to generate a clinically relevant target population – overlapped between groups. That is, a subject in the treatment group receives a weight that is the probability of not receiving the treatment (i.e., 1 – PS), while a subject in the control group receives a weight that is the probability of receiving the treatment (i.e., PS). As a consequence, the two groups have overlapped PS distributions. Those subjects overlapped between the two groups in the PS distribution receive more weight, while those who are only in one non-overlapping tail of the PS distribution receive less. Also OW does not prune any subjects. The target of inference, advantages, and disadvantages of the OW and FS methods are compared in eTable 1.

### PS-based fine stratification method (FS)

The FS method proposed by Desai et al. (2017) [8] finds matched balancing scores (PS) via stratification with a large number of PS strata (much larger than five in the traditional stratification method), and then assigns appropriate weights to subjects per stratum. It minimizes any loss of exposed subjects that may be relevant especially when treatment exposure is rare, because losing subjects decreases precision of the treatment effect estimates [8]. The method only excludes subjects whose PS are not in the overlapped PS regions between the two groups. There are two steps for implementation: (1) create equally-sized PS strata by ranking only treated/exposed subjects based on PS values and then assign control/unexposed subjects to these strata; (2) following stratification, in all strata with at least one treated patient and one control patient, weights are calculated (see below).

Regarding the optimum number of strata, Desai et al. stated that it may be difficult to make general recommendations because it may depend on the prevalence of a rare-exposed treatment [8]. The number of PS strata they used was 10, 50, or 100 and all produced similar bias and precisions in their simulations. In this study, we chose 20 PS strata, their stratification width about 0.05 on average, smaller than the recommended PS width of 0.2 [7]. . Each stratum had about 225 subjects from the FQHC-exposed group in our empirical example.

### Target of inference (estimand) and weights

In the study, since each patient can switch their primary care visits between FQHCs and non-FQHCs, we estimated ATE among all patients [6]. In literature, there are two existing approaches to assign weights for ATE. One approach is to generate equal total weights between groups, denoted as ‘ATE-equ,’ is based on *N*total in stratum i/*N*total exp in stratum i for the exposed group and N"total in stratum i"/N"total unexp in stratum i" for the unexposed group [18, 19]. The other approach, denoted as ‘ATE-unequ,’ is based on (*N*total in stratum i/*N*total)/(*N*total exp in stratum i/*N*total exp") for the exposed group and (*N*total in stratum i/*N*total)/(*N*total unexp in stratum i/*N*total unexp) for the unexposed group [6, 20]. This alternative approach results in the total weight in one group equivalent to the sample size in that group. The two weighting methods are very similar, except that ATE-unequ has a weight of N“total exp” (N“total unexp”) for the exposed (unexposed) group.

As a weighting method, OW targets the overlap population and its corresponding estimand is referred to as ATE on the overlap population (ATO) [12]. Zhou et al. (2020) stated that OW was part of a class of balancing weights that target a judiciously chosen subpopulation of interest from which an estimand is closely related to ATE [14]. Not surprisingly, OW’s total weights are identical between groups, the same as ATE-equ.

### Evaluation of performance via the empirical example

In the empirical study for the method evaluation [21], we used the standardized mean difference between the two groups (SMD), Mahalanobis balance (MB), and final sample size of retaining sample. SMD is a distance measure of balancing criterion for each covariate [22]. MB is a metric that measures the distance between two group mean vectors of all covariates and is standardized by the sample covariance matrix [1, 17, 23]. Final sample size [8] is a measure of model precision and can be important for a rare event outcome.

### Simulations

After balancing covariates, we determined relative performance of OW and FS for model bias and precision. The degree of covariate imbalance is proportional to bias in the treatment effect [24], and final sample size is associated with precision. However, due to lack of knowledge of the true FQHC effect in the empirical example, we do not know the real size of model bias and precision, especially, in a setting of infrequent exposure/outcome. Therefore, we conducted simulations.

Instead of using ordinary simulation approaches that do not capture important features that may exist between covariates, we chose the plasmode approach to conduct simulations [25–27]. Through resampling with replacement from all the observed covariates, plasmode can preserve the associations between covariates with potential complex covariance structures, which are common in healthcare claims databases [25]. Via a logistic regression model, details were provided in Appendix A (including R code) on how to simulate an outcome and/or an exposure factor. There were two logit models for outcome and exposure, respectively, with two different linear combinations of covariates. We simulated two types of treatment effects: homogeneity and heterogeneity. To simulate a heterogeneous treatment effect, we replaced constant treatment effect with an interaction term between exposure and sex (or age): sex, as an example, represented as a binary heterogeneity factor and age was a continuous one [25]. Age was standardized first before conducting a heterogeneous treatment effect. The simulation settings can be found in eTable 2.

To examine settings with infrequent outcome and/or occasional exposure, for each type of treatment effect, we simulated four scenarios by varying outcome risks and/or exposure prevalence. Scenarios simulated outcome risks of 1%, 10%, and 30% with the observed exposure prevalence (10.55%) or with 2.5% simulated exposure. We also simulated exposure prevalence of 2.5%, 10%, and 30% with the observed outcome risk (27.75%) or with 1% simulated outcome risk. We set the true FQHC effect to be one as a coefficient to both homogeneous and heterogeneous treatment terms. For each scenario, we simulated 500 datasets, each with the sample size of 4,000. For each simulated dataset, a weighted generalized linear model (GLM) with the log link function in the SAS GENMOD procedure was used to estimate the FQHC effect, i.e., natural logarithms of relative risk ratio [8]. Due to non-uniform weights included in our GLMs, instead of using the default delta method, we used the cluster-robust standard error method to estimate standard error (SE) of the effect [28, 29]. After covariates balanced, adjusting further for covariates is unnecessary because it is unrelated to the treatment independent variable [30]. That is, a simple difference in means on the balanced data can estimate the causal effect.

### Evaluation of performance in simulations

In the simulation study, we used the following criteria to evaluate the methods: mean MB, mean relative bias (rbias), standard deviation (SD) of rbias, square root of mean squared error of bias (rMSE), average SE of the estimated effect, average final sample size, two coverages [8, 17, 23], and significance. Relative bias is the percent relative difference, 100(estimated effect -truth)/truth [12–14]. The rMSE combines squared bias (not rbias) and its variance. The coverage is a probability of the 95% confidence interval (CI) that covers the true effect (denoted as ‘coverage’) [12, 13, 31]. It can be obtained with two steps: (1) to compute a CI via our weighted GLM and (2) then to calculate a proportion of samples covering the true effect among 500 simulations. In our simulation study, a CI could cover both the non-zero true effect and zero, and statistical significance may be influenced. Therefore, to distinguish from the traditional coverage, we generated another one (‘coverageT’) counting those CIs that cover the true effect but not zero. In some cases where CIs were too narrow to cover the true effect (see results below), significance was defined as a proportion of samples obtaining a significant effect (by a weighted GLM with a two-sided *p*-value < 0.05). The two coverages and significance are associated with model precision [32], but more targeted to detect the true treatment effect. Among the criteria, the least useful criterion is SE because it measures variability of effect in a model, not bias, precision, or measures in covariate balance.

### Unmatched subjects

Although matching was not involved in the study, via simulations we discovered whether pruning those clearly unmatched subjects has any effects on model bias and precision. The unmatched subjects are those who are available from one group but not from the other group in terms of combination cells of binary covariates.

### Summary of all methods

In summary, we used two datasets for performance evaluation: one was the original full dataset (denoted as ‘F’); the other was the dataset (denoted as ‘X’) after deleting those unmatched subjects. We also evaluated the two weighting approaches. Therefore, there were a total of seven methods for comparisons: crude, OW_F_, OW_X_, FS_F−equ_, FS_X−equ_, FS_F−uneq_, and FS_X−unequ_ (summary can be found in eTable 3). We used SAS version 9.4 to conduct covariate balancing, and statistical modeling for both empirical and simulation studies (Appendix A for analysis of one simulation), and we used R function (Appendix B) to generate simulation datasets in R version 4.3.0.

## Results

### Analysis of the empirical dataset

Table 1 shows the evaluation of the seven methods using the real-world data. OW_F_ and OW_X_ were nearly identical and performed the best by reducing all SMD of covariates to zero, and the smallest MB over all covariates, indicating perfect balancing of covariates. The four FS methods consistently performed fairly well over all covariates, with all SMD around zero. Among them, the two FS with equal weights between groups (FS_F−equ_ and FS_X−equ_) were closer to each other and achieved better MB than the other two FS with unequal weights (FS_F−unequ_ and FS_X−unequ_). The crude method exhibited the worst performance, far more imbalanced.

For the final sample size used for further analysis, OW_X_ excluded 147 subjects (0.345%) who had no matches for combinations of all binary variables. Using the full dataset, FS excluded 21 subjects (< 0.05%) whose PS were in non-overlapped regions. Distributions of PS per group were in eFigure 1.

### Analysis of simulated datasets with the homogeneous treatment effect

Tables 2 and 3 showed the simulation evaluation of each method by risk level of outcome and by exposure prevalence, respectively. In most scenarios, OW_F_ and OW_X_ had very similar results and both performed better than the other methods. That is, both OW had small MB, rbias, SD of rbias, rMSE, relatively small SE, and relatively large coverage and coverageT. There were two exceptions. One was that the crude method had smallest SE of estimate. The reason is that model estimation by the crude method is consistent with the simulation method (i.e., two logit models with a constant and additive treatment effect). The second exception was the cases with rare outcome events (1%) and low exposures (2.5% and 10%) (Table 2), where the crude method had the smallest rbias, SD of rbias, and rMSE compared to the others. The reason is that both rare events and low exposures resulted in complete separation or quasi-complete separation of data points that caused model estimation to be unstable [33, 34]. After removing these simulated samples, both OW had smaller rbias and larger coverage than the others (eTable 4).

**Table 1:** Evaluation of OW and FS in the empirical example

Covariates	Crude	OW_F_	OW_X_	FS_F−equ_	FS_X−equ_	FS_F−unequ_	FS_X−unequ_
	standardized mean difference (SMD)						
Age	9.35	0.00	0.00	-0.88	-0.90	-2.00	-2.04
Distance	-28.37	0.02	0.02	-1.15	-1.21	-2.73	-2.87
N of elig months	-24.86	0.01	0.01	1.01	0.96	2.27	2.15
N of MC months	-21.38	0.01	0.01	1.30	1.26	2.92	2.83
Elixhauser score	-19.78	0.00	0.00	-0.56	-0.57	-1.30	-1.33
Female	-8.47	0.00	0.00	0.79	0.70	0.79	0.70
Race group							
White	-15.25	0.00	0.00	-1.07	-1.05	-1.07	-1.05
Black	14.73	0.00	0.00	-1.83	-1.79	-1.83	-1.79
American Indian	-1.88	0.00	0.00	-0.76	-0.44	-0.76	-0.44
Asian	-7.66	0.00	0.00	-2.32	-2.29	-2.32	-2.29
Hispanic	-0.71	0.00	0.00	2.99	2.94	2.99	2.94
Unknown	4.36	0.00	0.00	0.3	0.22	0.28	0.22
Medicaid eligibility							
Blind/disabled	-2.51	0.00	0.00	-3.03	-3.16	-3.03	-3.16
Adult	2.51	0.00	0.00	3.03	3.16	3.03	3.16
TANF	1.40	0.00	0.00	3.09	3.26	3.09	3.26
Urban	7.76	0.01	0.00	0.94	1.06	0.94	1.06
Insulin	-3.94	0.00	0.00	1.32	1.38	1.32	1.38
**MB**	0.52	0.00033	0.00031	0.0498	0.0499	0.0714	0.0716
**N used**	42,628	42,628	42,481	42,607	42,459	42,607	42,459

Footnotes:

1. Crude = summarized by raw data without any balancing method; OW = overlap weighting method; FS = propensity score based fine stratification method

2. ‘F’ = a full set of data; ‘X’ = a subset of data after removing those unmatched

3. ‘equ’ = ATE with the equal weighting between groups; ‘unequ’ = ATE with the unequal weighting, where total weight in one group equivalent to the sample size in that group

4. MB = Mahalanobis balance.

5. N used = the total sample size that was used further for GLM analysis

6. TANF = temporary assistance for needy families

**Table 2:** Evaluation of OW and FS methods by simulation with the constant true effect by outcome risk along with observed/simulated exposure

Outcome risk	Methods	MB	rbias	SE	SD(rBias)	rMSE	Coverage	CoverageT	Significance	*N* used
Exposure prevalence = 10.55% (observed)										
1%	Crude	0.5542	-66.85	**0.470**	49.37	0.831	78.0	17.2	17.2	4000
	FS_F−equ_	0.0811	-25.04	0.498	51.46	0.572	96.0	41.0	41.8	3971
	FS_X−equ_	0.0760	-26.00	0.500	52.11	0.582	96.2	38.8	39.6	3812
	FS_F−unequ_	0.1146	-25.04	0.498	51.46	0.572	96.0	41.0	41.8	3971
	FS_X−unequ_	0.1098	-26.00	0.500	52.11	0.582	96.2	38.8	39.6	3812
	OW_F_	0.0006	**-7.69**	0.483	**47.79**	**0.484**	97.0	**52.4**	**54.8**	4000
	OW_X_	**0.0001**	-8.08	0.486	48.45	0.491	**97.2**	51.6	54.0	3835
10%	Crude	0.5524	-70.06	**0.136**	14.49	0.715	0.0	0.0	59.6	4000
	FS_F−equ_	0.0810	-47.74	0.146	12.95	0.495	5.2	5.2	94.0	3970
	FS_X−equ_	0.0761	-47.92	0.147	13.08	0.497	5.0	5.0	93.8	3815
	FS_F−unequ_	0.1145	-47.74	0.146	12.95	0.495	5.2	5.2	94.0	3970
	FS_X−unequ_	0.1100	-47.92	0.147	13.08	0.497	5.0	5.0	93.8	3815
	OW_F_	0.0006	**-39.30**	0.140	12.30	**0.412**	14.4	14.4	**99.6**	4000
	OW_X_	**0.0001**	-39.52	0.141	**12.28**	0.414	**14.6**	**14.6**	**99.6**	3839
30%	Crude	0.5526	-72.39	**0.067**	6.91	0.727	0.0	0.0	96.8	4000
	FS_F−equ_	0.0803	-61.44	0.071	6.18	0.617	0.0	0.0	**100.0**	3968
	FS_X−equ_	0.0759	-61.47	0.071	6.19	0.618	0.0	0.0	**100.0**	3814
	FS_F−unequ_	0.1135	-61.44	0.071	6.18	0.617	0.0	0.0	**100.0**	3968
	FS_X−unequ_	0.1096	-61.47	0.071	6.19	0.618	0.0	0.0	**100.0**	3814
	OW_F_	0.0006	**-56.17**	0.069	**6.05**	**0.565**	0.0	0.0	**100.0**	4000
	OW_X_	**0.0001**	-56.20	0.070	6.10	0.565	0.0	0.0	**100.0**	3840
**Exposure prevalence = 2.5%**										
1%	Crude	0.643	**-564.17**	**0.815**	**959.38**	**11.17**	**76.4**	10.0	33.6	4000
	FS_F−equ_	0.196	-689.45	0.849	1173.21	13.64	72.2	17.2	44.4	3859
	FS_X−equ_	0.188	-663.38	0.863	1157.41	13.36	72.4	15.4	41.8	3360
	FS_F−unequ_	0.291	-596.65	0.849	1005.94	11.69	72.2	17.2	44.4	3859
	FS_X−unequ_	0.293	-575.08	0.863	996.42	11.50	72.4	15.4	41.8	3360
	OW_F_	**0.031**	-606.14	0.825	1129.22	12.82	74.8	**24.0**	**49.2**	4000
	OW_X_	0.116	-614.94	0.836	1132.19	12.89	74.6	23.4	48.8	3459
10%	Crude	0.645	-73.23	**0.270**	27.75	0.78	13.2	13.2	24.8	4000
	FS_F−equ_	0.196	-48.49	0.319	30.44	0.57	76.0	42.4	42.6	3857
	FS_X−equ_	0.188	-48.96	0.323	31.65	0.58	76.0	42.0	42.2	3339
	FS_F−unequ_	0.291	-48.49	0.319	30.44	0.57	76.0	42.4	42.6	3857
	FS_X−unequ_	0.294	-48.96	0.323	31.65	0.58	76.0	42.0	42.2	3339
	OW_F_	**0.006**	**-40.89**	0.273	**22.97**	**0.47**	**78.4**	**61.0**	**63.4**	4000
	OW_X_	0.123	-42.62	0.277	35.88	0.56	77.6	59.6	61.6	3443
30%	Crude	0.645	-73.25	**0.131**	12.57	0.74	0.0	0.0	56.2	4000
	FS_F−equ_	0.191	-61.63	0.154	14.17	0.63	0.0	0.0	70.8	3860
	FS_X−equ_	0.186	-60.66	0.155	13.79	0.62	0.2	0.2	72.4	3355
	FS_F−unequ_	0.281	-61.63	0.154	14.17	0.63	0.0	0.0	70.8	3860
	FS_X−unequ_	0.287	-60.66	0.155	13.79	0.62	0.2	0.2	72.4	3355
	OW_F_	**0.030**	**-56.15**	0.132	**11.02**	**0.57**	0.0	0.0	**90.8**	4000
	OW_X_	0.031	-56.21	0.133	11.06	0.57	0.2	0.2	90.6	3454

Footnotes:

1. Crude = summarized by raw data without any balancing method; OW = overlap weighting method; FS = propensity score based fine stratification method

2. ‘F’ = a full set of data; ‘X’ = a subset of data after removing those unmatched

3. ‘equ’ = ATE with the equal weighting between groups; ‘unequ’ = ATE with the unequal weighting, where total weight in one group equivalent to the sample size in that group

4. The best values are bolded and can be used to guide which method performs the best per evaluation criterion

5. MB = Mahalanobis balance; rBias = relative bias = 100*(estimated effect – true effect) /true effect; SE = average estimated standard error; SD(rBias) = empirical standard deviation of relative bias x 100; rMSE = square root of mean squared error that combines squared bias (not relative bias) and its variance; Coverage = proportion of samples whose 95% CI cover the true effect; CoverageT = proportion of samples whose 95% CI cover the true effect but not zero; Significance = proportion of samples obtaining a significant effect (by a weighted GLM with a two-sided *p*-value < 0.05); N used = average total sample size that was used further for GLM.

**Table 3:** Evaluation of OW and FS methods by simulation with the constant true effect and by exposure prevalence with observed outcome risk or with simulated outcome risk of 1%

Exposure Prevalence	Methods	MB	rbias	SE	SD(rbias)	rMSE	Coverage	CoverageT	Significance	*N* used
Outcome risk = 27.75% (observed)										
2.5%	Crude	0.6434	-72.82	**0.139**	13.020	0.740	0.0	0.0	53.0	4000
	FS_F−equ_	0.1965	-60.23	0.163	14.365	0.619	0.4	0.4	69.6	3859
	FS_X−equ_	0.1884	-60.44	0.187	13.245	0.619	0.6	0.6	55.8	2801
	FS_F−unequ_	0.2906	-60.23	0.163	14.365	0.619	0.4	0.4	69.6	3859
	FS_X−unequ_	0.2932	-59.23	0.165	14.428	0.610	0.8	0.8	71.0	2801
	OW_F_	**0.0312**	**-54.61**	0.140	**12.077**	**0.559**	1.0	**1.0**	**89.0**	4000
	OW_X_	0.1551	-58.73	0.170	14.517	0.605	**1.2**	**1.0**	70.2	2883
10%	Crude	0.5556	-71.85	**0.073**	7.672	0.723	0.0	0.0	94.8	4000
	FS_F−equ_	0.0851	-60.21	0.077	6.703	0.606	0.0	0.0	99.8	3969
	FS_X−equ_	0.0783	-60.33	0.078	6.840	0.607	0.0	0.0	99.8	3804
	FS_F−unequ_	0.1195	-60.21	0.077	6.703	0.606	0.0	0.0	99.8	3969
	FS_X−unequ_	0.1131	-60.33	0.078	6.840	0.607	0.0	0.0	99.8	3804
	OW_F_	0.0008	**-54.77**	0.075	**6.666**	**0.552**	0.0	0.0	**100.0**	4000
	OW_X_	**0.0001**	-54.82	0.075	6.769	0.552	0.0	0.0	**100.0**	3828
30%	Crude	0.5173	-72.00	**0.050**	4.860	0.722	0.0	0.0	**100.0**	4000
	FS_F−equ_	0.0505	-59.43	0.051	4.167	0.596	0.0	0.0	**100.0**	3985
	FS_X−equ_	0.0480	-59.45	0.052	4.238	0.596	0.0	0.0	**100.0**	3925
	FS_F−unequ_	0.0710	-59.43	0.051	**4.167**	0.596	0.0	0.0	**100.0**	3985
	FS_X−unequ_	0.0677	-59.45	0.052	4.238	0.596	0.0	0.0	**100.0**	3925
	OW_F_	0.0003	**-55.83**	0.052	4.303	**0.560**	0.0	0.0	**100.0**	4000
	OW_X_	**0.0000**	-55.85	0.053	4.335	0.560	0.0	0.0	**100.0**	3938
Outcome risk = 1% *										
10%	Crude	0.5555	-76.04	**0.492**	**120.55**	1.426	79.2	17.0	17.2	4000
	FS_F−equ_	0.0831	-34.09	0.522	131.28	1.357	94.8	39.0	40.4	3967
	FS_X−equ_	0.0790	-36.08	0.525	131.62	1.365	95.8	36.6	37.8	3803
	FS_F−unequ_	0.1170	-33.69	0.522	123.27	**1.278**	94.8	39.0	40.4	3967
	FS_X−unequ_	0.1142	-35.77	0.525	123.63	1.287	95.8	36.6	37.8	3803
	OW_F_	0.0007	-16.84	0.505	129.39	1.305	96.4	**48.8**	**51.8**	4000
	OW_X_	**0.0001**	**-17.35**	0.508	129.60	1.308	**96.6**	48.0	51.0	3829
30%	Crude	0.5155	-63.22	**0.316**	33.09	0.714	47.4	22.4	22.4	4000
	FS_F−equ_	0.0487	-20.00	0.326	32.86	0.385	92.2	69.8	70.4	3985
	FS_X−equ_	0.0467	-21.63	0.328	**32.75**	0.392	91.8	67.2	67.4	3925
	FS_F−unequ_	0.0686	-20.00	0.326	32.86	0.385	92.2	69.8	70.4	3985
	FS_X−unequ_	0.0658	-21.63	0.328	32.75	0.392	91.8	67.2	67.4	3925
	OW_F_	0.0003	**-4.04**	0.330	32.95	**0.332**	**96.0**	80.2	82.0	4000
	OW_X_	**0.0000**	-4.75	0.332	33.22	0.336	**96.0**	**80.4**	**82.2**	3939

Footnotes:

*The simulation scenario with 1% outcome risk and 2.5% exposure prevalence is not shown here because it has been shown in Table 1

1. Crude = summarized by raw data without any balancing method; OW = overlap weighting method; FS = propensity score based fine stratification method

2. ‘F’ = a full set of data; ‘X’ = a subset of data after removing those unmatched

3. ‘equ’ = ATE with the equal weighting between groups; ‘unequ’ = ATE with the unequal weighting, where total weight in one group equivalent to the sample size in that group

4. The best values are bolded and can be used to guide which method performs the best per evaluation criterion

5. MB = Mahalanobis balance; rBias = relative bias = 100*(estimated effect – true effect) /true effect; SE = average estimated standard error; SD(rBias) = empirical standard deviation of relative bias x 100; rMSE = square root of mean squared error that combines squared bias (not relative bias) and its variance; Coverage = proportion of samples whose 95% CI cover the true effect; CoverageT = proportion of samples whose 95% CI cover the true effect but not zero; Significance = proportion of samples obtaining a significant effect (by a weighted GLM with a two-sided *p*-value < 0.05); N used = average total sample size that was used further for GLM.

Similar to the empirical study, the four FS methods were quite close to each other. The two FS with equal weights had smaller MB than the two FS with unequal weights. However, each pair of FS using the data with the same sample sizes (either full or reduced datasets) had almost the same model estimations. These indicate that the two ATE weighting methods had minor difference in balancing values but almost identical values in model estimation. The two FS using the full datasets generally had better model estimations than the two using the reduced datasets.

In the criteria, there were different change patterns over simulations. As outcome risk (Table 2) or exposure prevalence (Table 3) increased, the power increased, SE, SD, rMSE, and both coverages decreased, and significance increased. Coverages decreased as outcome risk (or exposure prevalence) increased because smaller SE resulted in a narrower CI of effect that were too narrow to cover the true effect. MB, unrelated to model estimation, remained stable in a method when outcome risk increased, and reduced greatly when exposure prevalence increased. On the other hand, rbias increased when outcome risk increased, and remained similar in a method when exposure increased.

To determine whether the simulation results were due to real differences or Monte Carlo error (MCE), we calculated MCE for both MB and rbias (eTables 5–6). We evaluated the number of simulations needed (Appendix B) and found that 500 simulations were enough for most settings of outcome and exposure.

### Analysis of simulated datasets with heterogeneous treatment effect

Tables 4 and 5 and eTables 7 and 8 showed the evaluation results due to sex(age)-dependent treatment effects. Similar to the results with the constant treatment effect, the two OW methods had very similar results and both performed better than the other methods in terms of MB, rbias, rMSE, coverages, and significance. Also the same to the homogeneous cases, there were two exceptions. One was that the crude method had smallest SE of estimate. The other was that FS had smaller rMSE than OW in the case with 1% outcome and 10% or 10.55% exposure. It was also due to the issue of complete separation or quasi-complete separation of data points in a few simulated samples. After removing those samples, the OW methods still performed the best (eTable 9). All change patterns across scenarios were consistent to those in the homogeneous cases.

**Table 4:** Evaluation of OW and FS methods by simulations with sex-dependent heterogeneous treatment effect by outcome risk along with observed/simulated exposure.*

Outcome risk	Methods	MB	rbias	SE	SD(rBias)	rMSE	Coverage	CoverageT	Significance	*N* used
Heterogeneity factor = sex and exposure prevalence = 10.55% (observed)										
1%	Crude	0.5499	-131.30	0.531	**372.130**	2.509	61.0	3.2	4.2	4000
	FS_F−equ_	0.0831	-61.15	0.557	379.639	**2.445**	92.8	20.2	21.2	3970
	FS_X−equ_	0.0778	-64.61	0.560	382.972	2.470	92.2	18.6	20.0	3815
	FS_F−unequ_	0.1170	-61.15	0.557	379.639	**2.445**	92.8	20.2	21.2	3970
	FS_X−unequ_	0.1121	-64.61	0.560	382.972	2.470	92.2	18.6	20.0	3815
	OW_F_	0.0007	**-41.72**	0.542	417.803	2.670	**97.6**	**28.6**	**30.0**	4000
	OW_X_	**0.0001**	-43.52	0.547	417.796	2.671	97.4	28.0	29.4	3838
10%	Crude	0.5502	-85.10	**0.149**	23.024	0.561	0.0	0.0	11.8	4000
	FS_F−equ_	0.0819	-44.47	0.159	21.119	0.313	0.0	0.0	62.4	3968
	FS_X−equ_	0.0754	-44.88	0.159	21.152	0.316	0.0	0.0	63.6	3811
	FS_F−unequ_	0.1153	-44.47	0.159	21.119	0.313	0.0	0.0	62.4	3968
	FS_X−unequ_	0.1087	-44.88	0.159	21.152	0.316	0.0	0.0	63.6	3811
	OW_F_	0.0007	**-36.06**	0.152	**20.265**	**0.263**	**0.4**	**0.4**	**77.6**	4000
	OW_X_	**0.0001**	-36.28	0.153	20.336	0.264	0.2	0.2	**77.6**	3836
30%	Crude	0.5552	-82.47	**0.074**	11.697	0.530	0.0	0.0	35.2	4000
	FS_F−equ_	0.0821	-61.37	0.078	10.583	0.396	0.0	0.0	88.0	3971
	FS_X−equ_	0.0770	-61.35	0.078	10.521	0.396	0.0	0.0	88.4	3816
	FS_F−unequ_	0.1157	-61.37	0.078	10.583	0.396	0.0	0.0	88.0	3971
	FS_X−unequ_	0.1114	-61.35	0.078	10.521	0.396	0.0	0.0	88.4	3816
	OW_F_	0.0007	**-56.94**	0.076	**10.124**	**0.368**	0.0	0.0	**96.2**	4000
	OW_X_	**0.0001**	-56.96	0.076	10.212	0.368	0.0	0.0	96.0	3841
**Heterogeneity factor = sex and exposure prevalence = 2.5%**										
10%	Crude	0.6445	-92.149	**0.301**	6.468	0.665	3.4	3.4	7.8	4000
	FS_F−equ_	0.1908	-48.554	0.352	7.558	0.459	58.2	20.4	20.6	3860
	FS_X−equ_	0.1855	-47.464	0.357	7.666	0.469	**60**	21.0	21.0	3355
	FS_F−unequ_	0.2808	-48.554	0.352	7.558	0.459	58.2	20.4	20.6	3860
	FS_X−unequ_	0.2867	-47.464	0.357	7.666	0.469	**60**	21.0	21.0	3355
	OW_F_	**0.0295**	-41.272	0.304	6.527	0.378	47.6	**26.6**	**27.8**	4000
	OW_X_	0.0311	**-40.999**	0.306	6.565	**0.376**	47.2	26.4	27.2	3454
30%	Crude	0.6535	-83.221	**0.145**	22.839	0.549	0.0	0.0	13.6	4000
	FS_F−equ_	0.1962	-59.869	0.170	22.499	0.407	0.0	0.0	36.2	3855
	FS_X−equ_	0.1848	-57.065	0.171	22.320	0.390	0.0	0.0	38.0	3347
	FS_F−unequ_	0.2904	-59.869	0.170	22.499	0.407	0.0	0.0	36.2	3855
	FS_X−unequ_	0.2882	-57.065	0.171	22.320	0.390	0.0	0.0	38.0	3347
	OW_F_	**0.0065**	**-56.260**	0.147	**18.623**	**0.377**	0.0	0.0	**49.6**	4000
	OW_X_	0.1147	-57.614	0.149	33.549	0.424	0.0	0.0	48.8	3449

Footnotes:

* The simulation scenario with 1% outcome risk and 2.5% exposure prevalence is not conducted due to both rare event and rare exposure that resulted in the issue of complete separation or quasi-complete separation of data points (shown in Table 2)

1. Crude = summarized by raw data without any balancing method; OW = overlap weighting method; FS = propensity score based fine stratification method

2. ‘F’ = a full set of data; ‘X’ = a subset of data after removing those unmatched

3. ‘equ’ = ATE with the equal weighting between groups; ‘unequ’ = ATE with the unequal weighting, where total weight in one group equivalent to the sample size in that group

4. The best values are bolded and can be used to guide which method performs the best per evaluation criterion

5. MB = Mahalanobis balance; The rbias, relative bias, was calculated as 100*(estimated effect – true effect)/true effect; SE = average estimated standard error; SD(rBias) = empirical standard deviation of relative bias x 100; rMSE = square root of mean squared error that combines squared bias (not relative bias) and its variance; Coverage = proportion of samples whose 95% CI cover the true effect; CoverageT = proportion of samples whose 95% CI cover the true effect but not zero; Significance = proportion of samples obtaining a significant effect (by a weighted GLM with a two-sided *p*-value < 0.05); N used = average total sample size that was used further for GLM.

6. The true sex-dependent treatment effect was 63.59%, calculated by the observed female proportion (63.59%) times true effect (= 1)

**Table 4:** Evaluation of OW and FS methods by simulation with the sex-dependent heterogeneous treatment effect by exposure prevalence with observed outcome risk or with simulated outcome risk of 1%

Exposure Prevalence	Methods	MB	rbias	SE	SD(rbias)	rMSE	Coverage	CoverageT	Significance	*N* used
Heterogeneity factor = sex and outcome risk = 27.75% (observed)										
2.5%	Crude	0.6492	-84.184	**0.154**	24.130	0.557	0	0	13.6	4000
	FS_F−equ_	0.1917	-60.292	0.181	23.414	0.411	0	0	31.0	3848
	FS_X−equ_	0.1873	-57.772	0.184	23.564	0.397	0	0	32.6	3336
	FS_F−unequ_	0.2832	-60.292	0.181	23.414	0.411	0	0	31.0	3848
	FS_X−unequ_	0.2916	-57.772	0.184	23.564	0.397	0	0	32.6	3336
	OW_F_	0.0418	-55.897	0.156	**19.064**	0.376	0	0	45.0	4000
	OW_X_	**0.0293**	**-55.757**	0.157	19.331	**0.375**	0	0	**45.4**	3450
10%	Crude	0.5543	-81.638	**0.080**	12.170	0.525	0	0	33.2	4000
	FS_F−equ_	0.0826	-58.987	0.084	11.299	0.382	0	0	87.6	3968
	FS_X−equ_	0.0776	-58.745	0.085	11.255	0.380	0	0	89.4	3804
	FS_F−unequ_	0.1171	-58.987	0.084	11.299	0.382	0	0	87.6	3968
	FS_X−unequ_	0.1127	-58.745	0.085	11.255	0.380	0	0	89.4	3804
	OW_F_	0.0008	-54.540	0.082	**10.949**	**0.354**	0	0	**95.8**	4000
	OW_X_	**0.0001**	**-54.538**	0.082	10.988	0.354	0	0	95.4	3829
30%	Crude	0.5133	-82.043	**0.053**	8.568	0.525	0	0	58.6	4000
	FS_F−equ_	0.0495	-58.906	0.054	7.183	0.377	0	0	99.8	3985
	FS_X−equ_	0.0478	-58.926	0.055	7.131	0.377	0	0	**100.0**	3926
	FS_F−unequ_	0.0697	-58.906	0.054	7.183	0.377	0	0	99.8	3985
	FS_X−unequ_	0.0674	-58.926	0.055	7.131	0.377	0	0	**100.0**	3926
	OW_F_	0.0003	-55.627	0.055	**7.295**	0.357	0	0	99.8	4000
	OW_X_	**0.0000**	**-55.606**	0.055	7.306	**0.357**	0	0	**100.0**	3939
Heterogeneity factor = sex and outcome risk = 1% *										
10%	Crude	0.5556	-121.79	**0.537**	**334.460**	2.263	60.2	6.6	7.4	4000
	FS_F−equ_	0.0832	-60.404	0.567	381.654	2.457	92.8	22.6	24.0	3968
	FS_X−equ_	0.0787	-55.555	0.571	344.830	**2.221**	92.2	22.6	24.0	3805
	FS_F−unequ_	0.1177	-60.404	0.567	381.654	2.457	92.8	22.6	24.0	3968
	FS_X−unequ_	0.1140	-55.555	0.571	344.830	**2.221**	92.2	22.6	24.0	3805
	OW_F_	0.0008	**-30.804**	0.548	374.815	2.391	**97.6**	**33.6**	**34.8**	4000
	OW_X_	**0.0001**	-31.930	0.552	374.932	2.393	**97.6**	31.6	32.8	3830
30%	Crude	0.5141	-85.691	**0.345**	59.821	0.665	23.4	5.8	6.4	4000
	FS_F−equ_	0.0495	-15.029	0.356	58.794	0.386	79.2	38.8	39.0	3984
	FS_X−equ_	0.0473	-17.597	0.358	59.135	0.392	76.6	37.6	37.8	3925
	FS_F−unequ_	0.0697	-15.029	0.356	58.794	0.386	79.2	38.8	39.0	3984
	FS_X−unequ_	0.0668	-17.597	0.358	59.135	0.392	76.6	37.6	37.8	3925
	OW_F_	0.0003	9.529	0.358	**58.189**	0.375	89.4	**54.0**	**54.2**	4000
	OW_X_	**0.0000**	**7.850**	0.361	58.524	**0.375**	**89.6**	52.8	52.8	3939

Footnotes:

1. Crude = summarized by raw data without any balancing method; OW = overlap weighting method; FS = propensity score based fine stratification method

2. ‘F’ = a full set of data; ‘X’ = a subset of data after removing those unmatched

3. ‘equ’ = ATE with the equal weighting between groups; ‘unequ’ = ATE with the unequal weighting, where total weight in one group equivalent to the sample size in that group

4. The best values are bolded and can be used to guide which method performs the best per evaluation criterion

5. MB = Mahalanobis balance; The rbias, relative bias, was calculated as 100*(estimated effect – true effect)/true effect; SE = average estimated standard error; SD(rBias) = empirical standard deviation of relative bias x 100; rMSE = square root of mean squared error that combines squared bias (not relative bias) and its variance; Coverage = proportion of samples whose 95% CI cover the true effect; CoverageT = proportion of samples whose 95% CI cover the true effect but not zero; Significance = proportion of samples obtaining a significant effect (by a weighted GLM with a two-sided *p*-value < 0.05); N used = average total sample size that was used further for GLM.

6. The true sex-dependent treatment effect was 63.59%, calculated by the observed female proportion (63.59%) times true effect (= 1)

## Discussion

Both OW and FS methods performed well among PS-based balancing methods for causal inference. To our knowledge, our study is the first to compare OW and FS as the two types of PS-based balancing methods: weighting and stratification. We used a real-world and simulated claims data for their relative performance. We included simulations of rare outcome and/or exposure, not rare in a claims-based observational study. We simulated data for both homogeneous and heterogeneous treatment effects. The OW method obtained nearly perfect covariate balance and performed much better in covariate balance, model bias, and model precision and coverages than FS.

The target of inference (estimand) we focused on was ATE due to the nature of the intervention in the real-world example where the intervention was feasible to treat all eligible patients. The target of inference by OW is a special ATE, called ATO. OW is part of a class of balancing weights that target a judiciously chosen subpopulation from which an estimand is closely related to ATE [14]. OW produces equal total weights between groups by its definition, i.e., making the two groups overlapped in terms of PS values. For the FS method, we evaluated the two published weighting algorithms for ATE estimation: with and without equal total weights between groups. We found that the ATE-equ performed better than ATE-unequ in terms of covariate balance (SMD and MB) but both algorithms had almost identical model estimation in terms of bias and precision. In its formula, compared with ATE-equ, ATE-unequ includes a group sample size in its numerator to a subject in that group. This additional piece was designed to normalize and stabilize weights by limiting unduly large weights [20]. However, the additional piece unequaled total weights between groups, reducing covariate balance slightly, but did not affect model bias and precision.

We assume that our study met all the key assumptions for causal inference, including the stable unit treatment value assumption, the consistency assumption, and the positivity assumption [14, 35]. However, practical violations of the positivity assumption occur when some subjects almost always (or almost never) receive treatment [14], for example, those unmatched in combinations of binary covariates. Our study explored if removing those unmatched helped covariate balance and model estimation. This was a minor matter in our case, maybe because the proportion of those removed was very low, about 0.34% of the whole population. Using the reduced data compared to using the full data, the simulation results showed slightly smaller covariate imbalance, but slightly larger model bias and imprecision. That is, although covariate balance is slightly reduced by allowing those clearly unmatched subjects between groups, larger sample size kept model estimation less biased and imprecise, especially with infrequent outcome and/or exposure. In addition, FS further removed some subjects with extreme PS, due to their PS not in the overlapped PS region between groups. However, comparing FS with OW which did not remove any subjects, we confidently state that given balanced PS values between groups, including mismatched subjects does not affect model estimation in settings with infrequent outcome/exposure.

In our weighted GLM analysis, we used the cluster-robust method to estimate SE of the intervention effect. It is inaccurate to use the delta method, the default model-based method, when using matching weights, because it assumes weights are frequency weights rather than probability weights [28]. 

In simulation results for both homogeneous and heterogeneous scenarios, we observed that as outcome risk level increased, bias increased. Higher risks and stronger correlations among exposure, outcome, and covariates led to larger bias in effect estimation [36]. That is, higher confounding, which we did not adjust for in analysis, caused more bias. Among 17 covariates, more than half of them were confounders, i.e., associated with hospitalization rate. As outcome risk increased, these confounders had more confounding effect that resulted in larger bias. Adjusting for those confounders could have improved model precision and accuracy. However, we purposely did not adjust further for them in the modeling stage because in the real-world example, investigators did not know which covariates were real confounders.

In simulation results, we also observed that as exposure prevalence increased, MB values in the crude method decreased. One possible reason is that higher exposure, and stronger correlations between covariates and exposure, resulted in more covariate balance. Furthermore, we found that as rate of outcome and/or exposure increased, coverages decreased and even became zero. That is, when there was larger power, CIs became too narrow to cover the true effect. Their 100% significance rate confirmed the reason.

The choices of our performance criteria were based on the guidance of metrics for covariate balance [21]. The MB criterion, which considers pairwise correlations between covariates, provides new insights beyond SMD. This is the first study to use MB to evaluate OW. In some simulation settings, coverage probability could be a maximum of 100% because it is different from confidence level [32]. Our study also solved the issue of some misleading results using the coverage probability as a criterion [14] by providing two coverages and one significance to replace the traditional one.

Besides OW, the FS method performed relatively well comparing the crude method. The FQHC and non-FQHC groups had significantly overlapped PS distributions, and only < 0.05% subjects were removed due to non-overlapped PS between them. Just as in Desai et al.’s study evaluating FS [8], after balancing covariates, the PS distributions became perfectly overlapped in the empirical example. This indicates that the number of strata, 20, was sufficient.

Our simulation results for constant treatment effect are consistent with Ripollone et al.’s study which also used simulated claims data [17]. In the simulated outcome with risk level of 20%, 20% exposure prevalence, and a sample size of 25,000, their FS analysis had 0.054 MB, 0.07–0.08 bias, and 0.178–0.172 rMSE, while ours had 0.047 MB, 0.0183 bias, and 0.025 rMSE, given the sample size of > 40,000 (eTable 10).

In our study, the two study groups were quite similar in that their PS distributions were substantially overlapped. However, when comparator groups are very different, the advantages of OW are actually greatest [37]. This is because the OW method will add more weight on those overlapped PS regions and fewer weights on those tailed PS regions. Given the same situation, the FS method will remove more subjects from non-overlapped regions which results in more severe bias and probably less model precision due to reduced sample size.

Our study has some limitations. First, the OW method can be used to estimate only ATE on the ATO population, but not average treatment effect on the treated population (ATT). However, two studies showed that when the exposure prevalence is small, ATO approximates ATT [12, 35]. Second, due to simulating rare outcome (1%) and exposure (2.5 -10.55%), some simulated samples faced the issue of complete separation or quasi-complete separation of data points that caused model estimation to be unstable. More advanced modeling methods could be used such as Firth’s method [34] and Bayesian method [33]. However, this is beyond the goal of the study. Third, our simulation findings may not be generalizable because our simulations were based on one empirical study. However, both OW and FS have been separately evaluated in multiple studies. Fourth, our simulation did not consider misspecifications of a PS model and/or degrees of overlap of PS distributions. However, Zhou et al [14] conducted simulations for such situations to compare the performances of OW, IPW, and other weighting methods. They found that OW was robust to these situations. One possible reason they pointed out was that the estimand of OW was not defined on the estimated but true PS and OW smoothly down-weights the influence of observations at both end of PS spectrum [14]. Last, to estimate PS, we used a logistic regression, that is, a logit modeled as a linear combination of covariates. To capture complex dependency patterns between outcome and covariates, a machine learning method such as random forest may provide more accurate and less model dependent estimate of PS [38]. This will be our future work.

## Conclusion

As demonstrated by our analysis with real-world and extensive simulated claims data, the OW method can yield nearly-perfect covariate balance while also retaining all of the sample. Therefore, OW can enhance the accuracy of ATE estimation over FS in most cases. Balancing covariates between treatment and control groups in observational studies can be challenging, especially in settings with infrequent outcomes and exposures. Both OW and FS methods can effectively balance covariates. These two different PS-based methods have been separately evaluated against other methods [8–14, 17] but have never been compared against each other. We found that OW generally led to better covariate balance and model precision. However, in settings with extremely rare outcomes (≤ 1%) and exposures (≤ 10%), OW performed slightly worse than FS in at least one evaluation criterion. Future studies should analyze scenarios with rare outcomes and exposures in more detail. In conclusion, OW could be considered an effective and easy-to-implement method for balancing covariates for ATE estimation in settings with infrequent but not too rare outcomes and exposures.

### Electronic supplementary material

Below is the link to the electronic supplementary material.

Supplementary Material 1

## Data Availability

Data are available from the Centers for Medicare & Medicaid Services (CMS) under data use agreement provisions. Per the data use agreement, the relevant limited datasets cannot be made publicly available. For any data request, please contact CMS via the link: https://www.cms.gov/Research-Statistics-Data-and-Systems/Computer-Data-and-Systems/MedicaidDataSourcesGenInfo/MAXGeneralInformation. After obtaining Taxes State Medicaid claims data in 2012, the definitions of study population with diabetes and the diabetes-related hospitalization outcome can be found in the main study published in Knitter et al. (2022, Medical Care) via the link: https://pubmed.ncbi.nlm.nih.gov/36040020/. The authors confirm that we all did not have any special access privileges that others would not have.

## Abbreviations

**Abbreviation**: **Meaning**
ATE: Average treatment effect in the total population
ATE-equ: The ATE method to generate equal total weights between groups
ATE-unequ: The ATE method to generate unequal total weights between groups
ATO: Average treatment effect on the overlap population
ATT: Average treatment effect on the treated population
CI: 95% confidence interval
Coverage: Probability of the 95% confidence interval that covers the true effect, ignoring whehter zero was covered or not
CoverageT: Probability of the 95% confidence interval (CI) that covers the true effect, but not zero
Crude: No balancing method used
FQHC: Federally qualified health centers
FS: Propensity score-based fine stratification method
FS_F−equ_: The FS method with a full set of data and subjects’ weights assigned by ATE-equ
FS_F−uneq_: The FS method with a full set of data and subjects’ weights assigned by ATE-unequ
FS_X−equ_: The FS method with a subset of data and subjects’ weights assigned by ATE-equ
FS_X−unequ_: The FS method with a subset of data and subjects’ weights assigned by ATE-unequ
IPW: Inverse probability weighting method
MB: Mahalanobis balance
OW: Propensity score-based Overlap weighting method
OW_F_: The OW method with a full set of data
OW_X_: The OW method with a subset of data
PS: Propensity score
PSM: Propensity score matching method
rbias: Mean relative bias
rMSE: Square root of mean squared error of bias
SD: Standard deviation of rbias
SMD: Standardized mean difference between the two groups
