# Supplementary material for: Comparison of two propensity score-based methods for balancing covariates: the overlap weighting and fine stratification methods in real-world claims data
Source: BMC Med Res Methodol. 2024 Jun 3;24:122. doi: 10.1186/s12874-024-02228-z (PMC11145799; doi:10.1186/s12874-024-02228-z)
Supplement: Supplementary file 1 — Supplementary Material 1 [file 12874_2024_2228_MOESM1_ESM.pdf]

Online Supplement for "Comparison of two propensity score-based methods for balancing covariates: the overlap weighting and fine stratification methods in real-world claims data"

eTable 1. Summary of the three balancing methods.

eTable 2. Observed associations of covariates with exposure or outcome in the empirical example and settings of simulations

eTable 3. Summary of the actual methods used for evaluation.

eTable 4. Evaluation of OW and FS methods over 500 simulations with true effect size = 1, sample size = 4000, outcome rate = 1%, exposure prevalence = 2.5% or 10%, but after removing simulation samples which had the issue of complete separation or quasi-complete separation of data points based on their GLM analyses.

eTable 5. Monte Carlo errors for MB and bias in simulations with the true effect size of one varied by outcome risk with observed exposure or with simulated exposure of 1% (for Table 2).

eTable 6. Monte Carlo errors for MB and bias in simulations with the true effect size of one varied by exposure prevalence with observed outcome risk or with simulated outcome risk of 1% (for Table 3).

eTable 7. Evaluation of OW and FS methods by simulations with age-dependent heterogeneous treatment effect by outcome risk along with observed/simulated exposure.*

eTable 8. Evaluation of OW and FS methods by simulation with the sex-dependent heterogeneous treatment effect by exposure prevalence with observed outcome risk or with simulated outcome risk of 1%.

eTable 9. Evaluation of OW and FS methods over 500 simulations with the heterogeneous treatment effects, sample size = 4000, outcome rate = 1%, exposure prevalence = 10.55% (observed) or 10% (simulated), but after removing those simulations which had the issue of quasi-complete separation of data points based on their GLM analyses.*

eTable 10. Evaluation of OW and FS methods over 500 simulations varied by exposure prevalence with true effect size = 0, sample size = 42,628, and observed outcome rate = 27.75%

eFigure 1. Plots of distributions of PS per group, associated with three methods: 1) upper left: crude; 2) upper right: FS1; 3) bottom: OW1.

Appendix A. SAS code for analysis of one simulated dataset

Appendix B. Implementation step of plasmode simulation

Appendix C. Evaluation of Number of Simulations

REFERENCES

eTable 1. Summary of the balancing methods.

Method	Type of balancing method	Target of inference	Weight calculation: treated	Weight calculation: control	Advantages	Disadvantages
**OW**	weighting	ATO	1-PS	PS	Avoid an issue of extreme weights. No subjects need to be excluded. Weights are appropriate in that more weight on those overlapped PS regions and fewer weights on those tailed PS regions.	Cannot be used for estimating ATT. Indirect weighting via PS
**FS**	stratification	ATE_{equ}	$N_{total\ in\ stratum\ i} / N_{total\ exp\ in\ stratum\ i}$	$N_{total\ in\ stratum\ i} / N_{total\ unexp\ in\ stratum\ i}$	No extreme weights. Instead of matching PS directly to calculate weights, based on quantiles of PS of the treated group, stratify PS with much more than 5 strata (say 20 in our study).	Those subjects whose PS are in non-overlapped regions need to be excluded. Indirect stratification via PS
		ATE_{unequ}	$(N_{total\ in\ stratum\ i}/N_{total})/(N\ N_{total\ exp\ in\ stratum\ i}/N_{total\ exp})$	$(N_{total\ in\ stratum\ i}/N_{total})/(N\ N_{total\ unexp\ in\ stratum\ i}/N_{total\ unexp})$		

Footnotes:

1. PS = propensity score;
2. ATE_{equ} = ATE with the equal total weights between groups;[1,2] ATE_{unequ} = ATE with the total weight in one group equivalent to the sample size in that group[3]

eTable 2. Observed associations of covariates with exposure or outcome in the empirical example and settings of simulations

	Exposure			Outcome (homogeneous)			Outcome (heterogenous - sex)	Outcome (heterogenous - age)
	Estimate	SE	P-value	Estimate	SE	P-value	Setting	Setting
**Intercept**	-0.255	0.192	0.183	-2.086	0.186	<.0001	-2.071	-2.046
**Age**	0.016	0.002	<.0001	-0.010	0.002	<.0001	-0.010	-0.010
**N of elig months**	-0.116	0.013	<.0001	-0.143	0.013	<.0001	-0.143	-0.144
**N of MC months**	-0.046	0.005	<.0001	-0.051	0.005	<.0001	-0.051	-0.051
**Elixhauser score**	-0.098	0.007	<.0001	0.751	0.008	<.0001	0.751	0.751
**Distance**	-0.016	0.001	<.0001	0.004	0.001	<.0001	0.004	0.004
**Female**	-0.124	0.033	0.000	-0.222	0.029	<.0001	-0.238	-0.222
**TANF**	-0.085	0.129	**0.512**	0.481	0.126	0.000	0.480	0.480
**Urban**	-0.252	0.052	<.0001	-0.060	0.043	**0.171**	-0.059	-0.060
**Insulin**	0.010	0.051	**0.843**	-0.273	0.043	<.0001	-0.272	-0.272
**Race group**								
**Unknown**	0.148	0.051	0.004	0.124	0.045	0.006	0.125	0.125
**White**	-0.107	0.045	0.017	0.146	0.036	<.0001	0.146	0.146
**Black**	0.347	0.042	<.0001	0.045	0.038	**0.238**	0.047	0.047
**American Indian**	-1.198	1.025	**0.243**	-0.080	0.626	**0.899**	-0.082	-0.084
**Asian**	-0.635	0.149	<.0001	-0.099	0.115	**0.391**	-0.101	-0.101
**Hispanic (reference)**								
**Medicaid Eligibility (Adult vs Blind/disabled)**	-0.022	0.131	**0.865**	0.256	0.128	0.046	0.256	0.256
**Exposure (FQHC vs non-FQHC)**				0.108	0.047	0.021	exposure*sex =1	exposure*(1+s_age) =1

Footnotes:

1. In a homogeneous treatment effect simulation model, the setting of each simulation were those estimated coefficients obtained by the observed data, except for the true exposure effect which was set to be 1.
2. In a heterogeneous treatment effect simulation model, its treatment effect depended on sex (or age) by using an interaction term between exposure and sex (age). For the age-dependent treatment simulation model, we first scaled age by standardizing it (denoted as 's_age') and then added one to avoid zero treatment effect.
3. The true effect in the covariate-dependent treatment effect was calculated to be mean covariate times true effect (=1). Therefore, the true effect of sex-dependent treatment was 63.59%, the observed proportion of female in the empirical example. The true effect of age-dependent treatment was 1.

eTable 3. Summary of the actual methods used for evaluation.

Type of balancing	Methods	Datasets used in both empirical and simulation studies
weighting	**OW_F**	full dataset
	**OW_X**	dataset after removing unmatched subjects based on combinations of binary covariates
stratification	**$FS_{F\text{-}equ}$**	full dataset by the ATE weighting approach with equal total weights between groups
	**$FS_{X\text{-}equ}$**	dataset after removing unmatched subjects based on combinations of binary covariates by the ATE weighting approach with equal total weights between groups
	**$FS_{F\text{-}unequ}$**	full dataset by the ATE weighting approach with unequal total weights (i.e., total weight equivalent to its sample size in that group)
	**$FS_{X\text{-}unequ}$**	datasets after removing unmatched subjects based on combinations of binary covariates by the ATE weighting approach with unequal total weights (i.e., total weight equivalent to its sample size in that group)

eTable 4. Evaluation of OW and FS methods over 500 simulations with true effect size = 1, sample size = 4000, outcome rate = 1%, exposure prevalence = 2.5% or 10%, but after removing simulation samples which had the issue of complete separation or quasi-complete separation of data points based on their GLM analyses.

**Methods**	**rbias**	**SE**	**SD(rBias)**	**rMSE**	**Coverage**	**CoverageT**	**Significance**
**Outcome = 1% and exposure prevalence =2.5%***							
Crude	-43.093	**0.815**	51.326	0.670	**99.74**	13.05	13.32
$FS_{F\text{-}equ}$	-91.722	0.849	488.402	4.969	94.26	22.45	27.42
$FS_{X\text{-}equ}$	-57.458	0.863	389.862	3.941	94.52	20.10	24.02
$FS_{F\text{-}unequ}$	-78.667	0.849	419.505	4.268	94.26	22.45	27.42
$FS_{X\text{-}unequ}$	-49.364	0.863	335.525	3.391	94.52	20.10	24.02
OW_F	15.780	0.825	**51.214**	**0.536**	97.65	**31.33**	**33.68**
OW_X	**5.034**	0.836	149.670	1.498	97.39	30.55	33.16
**Outcome = 1% and exposure prevalence =10%****							
Crude	-71.302	**0.492**	54.165	0.895	79.359	17.0	17.0
$FS_{F\text{-}equ}$	-28.883	0.522	56.900	0.638	94.990	39.1	40.3
$FS_{X\text{-}equ}$	-30.869	0.525	57.424	0.652	95.992	36.7	37.7
$FS_{F\text{-}unequ}$	-28.883	0.522	56.900	0.638	94.990	39.1	40.3
$FS_{X\text{-}unequ}$	-30.869	0.525	57.424	0.652	95.992	36.7	37.7
OW_F	**-11.620**	0.505	**51.807**	**0.531**	96.593	**48.9**	**51.7**
OW_X	-12.123	0.508	52.168	0.536	**96.794**	48.1	50.9

Footnotes:

* There were a total of 383 simulated samples for the summary by excluding 117 samples which had the issue of complete separation or quasi-complete separation of data points.

**There were a total of 499 simulated samples for the summary by excluding one sample which had quasi-complete separation of data points.

eTable 5. Monte Carlo errors for MB and bias in simulations with the true effect size of one varied by outcome risk with observed exposure or with simulated exposure of 1% (for Table 2).

Outcome risk	Methods	MB	MCE(MB)	rbias	MCE(rbias)
**Exposure prevalence = 10.55% (observed)**					
1%	Crude	0.5542	0.0022	-66.85	2.206
	$FS_{F\text{-}equ}$	0.0811	0.0008	-25.04	2.299
	$FS_{X\text{-}equ}$	0.0760	0.0007	-26.00	2.328
	$FS_{F\text{-}unequ}$	0.1146	0.0011	-25.04	2.299
	$FS_{X\text{-}unequ}$	0.1098	0.0011	-26.00	2.328
	OW_F	0.0006	0.0000	**-7.69**	2.135
	OW_X	**0.0001**	0.0000	-8.08	2.165
10%	Crude	0.5524	0.0023	-70.06	0.647
	$FS_{F\text{-}equ}$	0.0810	0.0008	-47.74	0.579
	$FS_{X\text{-}equ}$	0.0761	0.0007	-47.92	0.584
	$FS_{F\text{-}unequ}$	0.1145	0.0010	-47.74	0.579
	$FS_{X\text{-}unequ}$	0.1100	0.0010	-47.92	0.584
	OW_F	0.0006	0.0000	**-39.30**	0.549
	OW_X	**0.0001**	0.0000	-39.52	0.549
30%	Crude	0.5526	0.0023	-72.39	0.309
	$FS_{F\text{-}equ}$	0.0803	0.0008	-61.44	0.276
	$FS_{X\text{-}equ}$	0.0759	0.0007	-61.47	0.277
	$FS_{F\text{-}unequ}$	0.1135	0.0011	-61.44	0.276
	$FS_{X\text{-}unequ}$	0.1096	0.0011	-61.47	0.277
	OW_F	0.0006	0.0000	**-56.17**	0.270
	OW_X	**0.0001**	0.0000	-56.20	0.273
**Exposure prevalence = 2.5%**					
1%	Crude	0.643	0.0049	**-564.17**	42.862
	$FS_{F\text{-}equ}$	0.196	0.0022	-689.45	52.415
	$FS_{X\text{-}equ}$	0.188	0.0022	-663.38	51.709
	$FS_{F\text{-}unequ}$	0.291	0.0039	-596.65	44.942
	$FS_{X\text{-}unequ}$	0.293159	0.0045	-575.08	44.517
	OW_F	**0.031**	0.0174	-606.14	50.450
	OW_X	0.116	0.0589	-614.94	50.583
10%	Crude	0.645	0.0045	-73.23	1.240
	$FS_{F\text{-}equ}$	0.196	0.0023	-48.49	1.360
	$FS_{X\text{-}equ}$	0.188	0.0021	-48.96	1.414
	$FS_{F\text{-}unequ}$	0.291	0.0039	-48.49	1.360
	$FS_{X\text{-}unequ}$	0.293727	0.0046	-48.96	1.414
	OW_F	**0.006**	0.0003	**-40.89**	1.026
	OW_X	0.123	0.0545	-42.62	1.603
30%	Crude	0.645	0.0049	-73.25	0.562
	$FS_{F\text{-}equ}$	0.191	0.0022	-61.63	0.633
	$FS_{X\text{-}equ}$	0.186	0.0020	-60.66	0.616
	$FS_{F\text{-}unequ}$	0.281	0.0037	-61.63	0.633
	$FS_{X\text{-}unequ}$	0.286694	0.0044	-60.66	0.616
	OW_F	**0.030**	0.0163	**-56.15**	0.492
	OW_X	0.031	0.0183	-56.21	0.494

**Mean**	0.0056	8.874
**Max**	0.0589	52.415
**Marge of error**	0.0700	4.500
**N based on mean**	0.0248	14.941
**N based on max**	2.7234	521.197

eTable 6. Monte Carlo errors for MB and bias in simulations with the true effect size of one varied by exposure prevalence with observed outcome risk or with simulated outcome risk of 1% (for Table 3)

Exposure Prevalence	Methods	MB	MCE(MB)	rbias	MCE(rbias)
**Outcome risk = 27.75% (observed)**					
2.5%	Crude	0.6434	0.0049	-72.82	0.5817
	$FS_{F\text{-}equ}$	0.1965	0.0022	-60.23	0.6418
	$FS_{X\text{-}equ}$	0.1884	0.0024	-60.44	0.5918
	$FS_{F\text{-}unequ}$	0.2906	0.0039	-60.23	0.6418
	$FS_{X\text{-}unequ}$	0.2932	0.0045	-59.23	0.6446
	OW_F	**0.0312**	0.0174	**-54.61**	0.5396
	OW_X	0.1551	0.0663	-58.73	0.6486
10%	Crude	0.5556	0.0025	-71.85	0.3428
	$FS_{F\text{-}equ}$	0.0851	0.0008	-60.21	0.2995
	$FS_{X\text{-}equ}$	0.0783	0.0007	-60.33	0.3056
	$FS_{F\text{-}unequ}$	0.1195	0.0011	-60.21	0.2995
	$FS_{X\text{-}unequ}$	0.1131	0.0011	-60.33	0.3056
	OW_F	0.0008	0.0000	**-54.77**	0.2978
	OW_X	**0.0001**	0.0000	-54.82	0.3024
30%	Crude	0.5173	0.0014	-72.00	0.2171
	$FS_{F\text{-}equ}$	0.0505	0.0005	-59.43	0.1862
	$FS_{X\text{-}equ}$	0.0480	0.0005	-59.45	0.1893
	$FS_{F\text{-}unequ}$	0.0710	0.0007	-59.43	0.1862
	$FS_{X\text{-}unequ}$	0.0677	0.0007	-59.45	0.1893
	OW_F	0.0003	0.0000	**-55.83**	0.1922
	OW_X	**0.0000**	0.0000	-55.85	0.1937
**Outcome risk = 1% ***					
10%	Crude	0.5555	0.0025	-76.04	5.3857
	$FS_{F\text{-}equ}$	0.0831	0.0008	-34.09	5.8649
	$FS_{X\text{-}equ}$	0.0790	0.0007	-36.08	5.8804
	$FS_{F\text{-}unequ}$	0.1170	0.0011	-33.69	5.5074
	$FS_{X\text{-}unequ}$	0.1142	0.0010	-35.77	5.5235
	OW_F	0.0007	0.0000	-16.84	5.7807
	OW_X	**0.0001**	0.0000	**-17.35**	5.7900
30%	Crude	0.5155	0.0015	-63.22	1.4785
	$FS_{F\text{-}equ}$	0.0487	0.0005	-20.00	1.4681
	$FS_{X\text{-}equ}$	0.0467	0.0005	-21.63	1.4634
	$FS_{F\text{-}unequ}$	0.0686	0.0007	-20.00	1.4681
	$FS_{X\text{-}unequ}$	0.0658	0.0007	-21.63	1.4634
	OW_F	0.0003	0.0000	**-4.04**	1.4723
	OW_X	**0.0000**	0.0000	-4.75	1.4843
**Mean**			0.00348		1.65222
**Max**			0.06632		5.88042
**Marge of error**			0.0400		4.0000
**N based on mean**			0.0290		0.6554
**N based on max**			10.5611		8.3025

eTable 7. Evaluation of OW and FS methods by simulations with age-dependent heterogeneous treatment effect by outcome risk along with observed/simulated exposure.*

Outcome risk	Methods	MB	rbias	SE	SD(rBias)	rMSE	Coverage	CoverageT	Significance	N used
**Heterogeneity factor = age and exposure prevalence = 10.55% (observed)**										
1%	Crude	0.5512	-53.73	**0.440**	**114.813**	1.268	89.0	27.0	27.2	4000
	FS_{F-equ}	0.0818	-16.20	0.469	120.058	1.211	**97.2**	51.6	53.6	3971
	FS_{X-equ}	0.0763	-16.13	0.471	119.940	**1.210**	**97.2**	51.4	53.2	3817
	$FS_{F-unequ}$	0.1155	-16.20	0.469	120.058	1.211	**97.2**	51.6	53.6	3971
	$FS_{X-unequ}$	0.1100	-16.13	0.471	119.940	**1.210**	**97.2**	51.4	53.2	3817
	OW_F	0.0007	6.25	0.453	126.783	1.269	94.0	**64.4**	**70.2**	4000
	OW_X	**0.0001**	**5.80**	0.457	126.879	1.270	94.2	63.8	69.4	3841
10%	Crude	0.5557	-58.36	**0.130**	13.145	0.598	0.2	0.2	85.2	4000
	FS_{F-equ}	0.0809	-40.35	0.141	12.248	0.422	11.6	11.6	99.4	3970
	FS_{X-equ}	0.0752	-40.08	0.141	12.375	0.419	11.8	11.8	98.8	3812
	$FS_{F-unequ}$	0.1141	-40.35	0.141	12.248	0.422	11.6	11.6	99.4	3970
	$FS_{X-unequ}$	0.1083	-40.08	0.141	12.375	0.419	11.8	11.8	98.8	3812
	OW_F	0.0007	-27.69	0.134	**11.599**	0.300	**46.0**	**46.0**	**100.0**	4000
	OW_X	**0.0001**	**-27.67**	0.135	11.634	**0.300**	45.2	45.2	**100.0**	3835
30%	Crude	0.5495	-65.01	**0.064**	6.352	0.653	0.0	0.0	100.0	4000
	FS_{F-equ}	0.0807	-58.46	0.070	5.858	0.588	0.0	0.0	100.0	3969
	FS_{X-equ}	0.0760	-58.04	0.070	**5.856**	0.583	0.0	0.0	100.0	3814
	$FS_{F-unequ}$	0.1141	-58.46	0.070	5.858	0.588	0.0	0.0	100.0	3969
	$FS_{X-unequ}$	0.1097	-58.04	0.070	**5.856**	0.583	0.0	0.0	100.0	3814
	OW_F	0.0007	-49.45	0.067	6.007	0.498	0.0	0.0	100.0	4000
	OW_X	**0.0001**	**-49.40**	0.067	6.004	**0.498**	0.0	0.0	100.0	3839
**Heterogeneity factor = age and exposure prevalence = 2.5%**										
10%	Crude	0.645	-63.165	0.258	26.753	0.686	25.6	25.6	36.6	4000
	FS_{F-equ}	0.191	-40.082	0.306	28.595	0.492	83.8	54.6	54.6	3860
	FS_{X-equ}	0.186	-39.785	0.310	29.222	0.494	83.4	57.8	57.8	3355
	$FS_{F-unequ}$	0.281	-40.082	0.306	28.595	0.492	83.8	54.6	54.6	3860
	$FS_{X-unequ}$	0.287	-39.785	0.310	29.222	0.494	83.4	57.8	57.8	3355
	OW_F	**0.030**	**-30.470**	**0.261**	**23.362**	**0.384**	**86.2**	**74.2**	**75.4**	4000
	OW_X	0.031	-30.485	0.263	23.449	0.385	85.2	73.2	74.2	3454
30%	Crude	0.653	-67.799	**0.126**	13.206	0.691	0.0	0.0	70.8	4000
	FS_{F-equ}	0.196	-56.625	0.149	14.327	0.584	0.4	0.4	81.2	3855
	FS_{X-equ}	0.185	-55.153	0.150	13.826	0.569	0.6	0.6	82.4	3347
	$FS_{F-unequ}$	0.290	-56.625	0.149	14.327	0.584	0.4	0.4	81.2	3855
	$FS_{X-unequ}$	0.288	-55.153	0.150	13.826	0.569	0.6	0.6	82.4	3347
	OW_F	**0.007**	**-50.424**	0.128	**12.382**	**0.519**	1.6	1.6	**96.2**	4000
	OW_X	0.115	-50.684	0.130	14.165	0.526	**2.0**	**2.0**	95.6	3449

Footnote:

*The simulation scenario with 1% outcome risk and 2.5% exposure prevalence was not conducted due to complete separation or quasi-complete separation of data points that caused model estimation to be unstable (see Table 2 and results section).

eTable 8. Evaluation of OW and FS methods by simulation with the sex-dependent heterogeneous treatment effect by exposure prevalence with observed outcome risk or with simulated outcome risk of 1%.

Exposure Prevalence	Methods	MB	rbias	SE	SD(rbias)	rMSE	Coverage	Coverage T	Significance	N used
**Heterogeneity factor = age and outcome risk = 27.75% (observed)**										
2.5%	Crude	0.649	-66.823	**0.133**	12.929	0.681	0.0	0.0	68.4	4000
	$FS_{F\text{-}equ}$	0.192	-55.162	0.157	13.760	0.569	0.6	0.6	81.0	3848
	$FS_{X\text{-}equ}$	0.187	-53.673	0.159	13.795	0.554	1.0	1.0	82.0	3336
	$FS_{F\text{-}unequ}$	0.283	-55.162	0.157	13.760	0.569	0.6	0.6	81.0	3848
	$FS_{X\text{-}unequ}$	0.292	-53.673	0.159	13.795	0.554	1.0	1.0	82.0	3336
	OW_F	**0.042**	-48.580	0.135	**11.264**	0.499	2.8	2.8	**98.2**	4000
	OW_X	0.029	**-48.495**	0.136	11.526	**0.498**	**3.4**	**3.4**	97.8	3450
10%	Crude	0.554	-66.384	**0.071**	7.043	0.668	0.0	0.0	99.6	4000
	$FS_{F\text{-}equ}$	0.083	-55.844	0.075	6.322	0.562	0.0	0.0	100.0	3968
	$FS_{X\text{-}equ}$	0.078	-55.848	0.075	6.364	0.562	0.0	0.0	100.0	3804
	$FS_{F\text{-}unequ}$	0.117	-55.844	0.075	6.322	0.562	0.0	0.0	100.0	3968
	$FS_{X\text{-}unequ}$	0.113	-55.848	0.075	6.364	0.562	0.0	0.0	100.0	3804
	OW_F	0.001	-49.406	0.073	**6.249**	**0.498**	0.0	0.0	100.0	4000
	OW_X	**0.000**	-49.484	0.073	6.310	0.499	0.0	0.0	100.0	3829
30%	Crude	0.513	-66.615	0.049	4.894	0.668	0.0	0.0	100.0	4000
	$FS_{F\text{-}equ}$	0.050	-54.859	0.050	4.383	0.550	0.0	0.0	100.0	3985
	$FS_{X\text{-}equ}$	0.048	-54.867	0.051	**4.365**	0.550	0.0	0.0	100.0	3926
	$FS_{F\text{-}unequ}$	0.070	-54.859	0.050	4.383	0.550	0.0	0.0	100.0	3985
	$FS_{X\text{-}unequ}$	0.067	-54.867	0.051	**4.365**	0.550	0.0	0.0	100.0	3926
	OW_F	0.000	**-50.953**	0.051	4.522	**0.512**	0.0	0.0	100.0	4000
	OW_X	**0.000**	-50.968	0.052	4.531	0.512	0.0	0.0	100.0	3939
**Heterogeneity factor = age and outcome risk = 1% ***										
10%	Crude	0.556	-55.827	**0.451**	**115.351**	1.282	86.6	27.2	27.6	4000
	$FS_{F\text{-}equ}$	0.083	-14.472	0.479	118.613	1.195	95.4	50.6	53.4	3968
	$FS_{X\text{-}equ}$	0.079	-15.073	0.482	118.350	**1.193**	**96**	50.4	52.8	3805
	$FS_{F\text{-}unequ}$	0.118	-14.472	0.479	118.613	1.195	95.4	50.6	53.4	3968
	$FS_{X\text{-}unequ}$	0.114	-15.073	0.482	118.350	**1.193**	**96**	50.4	52.8	3805
	OW_F	0.001	4.288	0.464	128.310	1.284	94.4	**63.0**	**68.4**	4000
	OW_X	**0.000**	**3.564**	0.469	128.723	1.288	94.6	62.0	67.2	3830
30%	Crude	0.514	-45.386	**0.307**	34.688	0.571	65.8	43.0	43.2	4000
	$FS_{F\text{-}equ}$	0.050	**-3.514**	0.317	33.958	**0.341**	95	82.6	85.0	3984
	$FS_{X\text{-}equ}$	0.047	-4.749	0.319	34.257	0.346	**95.4**	82.2	84.0	3925
	$FS_{F\text{-}unequ}$	0.070	**-3.514**	0.317	33.958	**0.341**	95	82.6	85.0	3984
	$FS_{X\text{-}unequ}$	0.067	-4.749	0.319	34.257	0.346	**95.4**	82.2	84.0	3925
	OW_F	0.000	13.845	0.322	34.196	0.369	92.2	**85.6**	**93.0**	4000
	OW_X	**0.000**	13.148	0.324	34.322	0.368	92.4	85.4	92.2	3939

eTable 9. Evaluation of OW and FS methods over 500 simulations with the heterogeneous treatment effects, sample size = 4000, outcome rate = 1%, exposure prevalence = 10.55% (observed) or 10% (simulated), but after removing those simulations which had the issue of quasi-complete separation of data points based on their GLM analyses.*

Outcome risk	Methods	rbias	SE	SD(rBias)	rMSE	Coverage	CoverageT	Significance	N of simulated samples
**Heterogeneity factor = sex and exposure prevalence =10.55% (observed)**									
	Crude	-94.816	**0.531**	**80.443**	0.791	61.0	3.2	3.2	495
	$FS_{F\text{-equ}}$	-24.048	0.557	87.449	0.577	92.8	20.2	20.2	495
	$FS_{X\text{-equ}}$	-27.201	0.560	89.012	0.592	92.2	18.6	19.0	495
1%	$FS_{F\text{-unequ}}$	-24.048	0.557	87.449	0.577	92.8	20.2	20.2	495
	$FS_{X\text{-unequ}}$	-27.201	0.560	89.012	0.592	92.2	18.6	19.0	495
	OW_F	**-0.558**	0.542	80.648	**0.513**	**97.6**	**28.6**	**29.0**	495
	OW_X	-2.384	0.547	81.966	0.521	97.4	28.0	28.4	495
**Heterogeneity factor = sex and exposure prevalence =10%**									
	Crude	-92.750	**0.537**	84.334	0.797	60.7	6.7	6.7	496
	$FS_{F\text{-equ}}$	-23.193	0.567	91.477	0.600	93.5	22.8	23.2	495
	$FS_{X\text{-equ}}$	-25.786	0.571	94.082	0.620	92.9	22.8	23.4	496
1%	$FS_{F\text{-unequ}}$	-23.193	0.567	91.477	0.600	93.5	22.8	23.2	495
	$FS_{X\text{-unequ}}$	-25.786	0.571	94.082	0.620	92.9	22.8	23.4	496
	OW_F	1.999	0.548	**82.741**	**0.526**	**98.4**	**33.9**	**34.3**	496
	OW_X	**0.877**	0.552	83.061	0.528	98.4	31.9	32.3	496
**Heterogeneity factor = age and Exposure prevalence =10.55% (observed)**									
	Crude	-48.996	**0.440**	44.594	0.663	89.0	27.0	27.0	499
	$FS_{F\text{-equ}}$	-11.230	0.469	45.551	0.469	**97.2**	51.6	53.4	499
	$FS_{X\text{-equ}}$	-11.166	0.471	45.521	0.469	**97.2**	51.4	53.0	499
1%	$FS_{F\text{-unequ}}$	-11.230	0.469	45.551	0.469	**97.2**	51.6	53.4	499
	$FS_{X\text{-unequ}}$	-11.166	0.471	45.521	0.469	**97.2**	51.4	53.0	499
	OW_F	11.552	0.453	45.221	0.467	94.0	**64.4**	**70.0**	499
	OW_X	**11.107**	0.457	**45.203**	**0.465**	94.2	63.8	69.2	499
**Heterogeneity factor = age and Exposure prevalence =10%**									
	Crude	-51.112	0.451	46.828	0.693	86.8	27.3	27.5	499
	$FS_{F\text{-equ}}$	-9.680	0.479	50.945	0.519	95.6	50.7	53.3	499
	$FS_{X\text{-equ}}$	-10.281	0.482	50.320	0.514	96.2	50.5	52.7	499
1%	$FS_{F\text{-unequ}}$	-9.680	0.479	50.945	0.519	95.6	50.7	53.3	499
	$FS_{X\text{-unequ}}$	-10.281	0.482	50.320	0.514	96.2	50.5	52.7	499
	OW_F	9.628	0.464	47.001	**0.480**	94.6	**63.1**	**68.3**	499
	OW_X	8.910	0.469	47.798	0.486	**94.8**	62.1	67.1	499

* The last column indicated a total of simulated samples used for the summary after removing those samples which had the issue of quasi-complete separation of data points.

eTable 10. Evaluation of OW and FS methods over 500 simulations varied by exposure prevalence with true effect size = 0, sample size = 42,628, and observed outcome rate = 27.75%*

Outcome risk	Methods	MB	Bias[a]	SE(Bias)[c]	rMSE	Coverage[b]	N used
1%	Crude	0.5651	-0.1756	0.088	0.1945	47.6	**42,628**
	$FS_{F\text{-}equ}$	0.0927	-0.0219	0.010	0.0635	23	42,301
	$FS_{X\text{-}equ}$	0.0893	-0.0211	0.010	**0.0622**	20.2	40,509
	$FS_{F\text{-}unequ}$	0.1318	-0.0219	0.074	0.0772	48.2	42,301
	OW_F	**0.0021**	**-0.0047**	0.124	0.139	**99.8**	**42,628**
	OW_X	0.0658	-0.0051	0.124	0.1391	**99.8**	40,762
5%	Crude	0.5353	-0.1693	0.040	0.1741	1	**42,628**
	$FS_{F\text{-}equ}$	0.0605	-0.0203	0.010	0.0357	39.8	42,536
	$FS_{X\text{-}equ}$	0.0588	-0.0199	0.010	**0.0357**	41	42,060
	$FS_{F\text{-}unequ}$	0.0862	-0.0203	0.035	0.0408	46.7	42,536
	OW_F	0.0005	0.0017	0.057	0.0644	**100**	**42,628**
	OW_X	**0**	**0.0014**	0.057	0.0644	**100**	42,136
10%	Crude	0.5243	-0.1675	0.028	0.17	0	**42,628**
	$FS_{F\text{-}equ}$	0.0535	-0.017	0.010	**0.0278**	53.2	42,570
	$FS_{X\text{-}equ}$	0.0526	-0.0172	0.010	0.0278	54	42,297
	$FS_{F\text{-}unequ}$	0.0763	-0.017	0.024	0.0301	46.4	42,570
	OW_F	0.0003	0.0021	0.041	0.0465	**100**	**42,628**
	OW_X	**0.0001**	**0.0018**	0.041	0.0465	**100**	42,345
20%	Crude	0.5144	-0.1682	0.020	0.1695	0	**42,628**
	$FS_{F\text{-}equ}$	0.048	-0.018	0.010	**0.0243**	59.8	42,592
	$FS_{X\text{-}equ}$	0.047	-0.0183	0.010	0.0245	58	42,416
	$FS_{F\text{-}unequ}$	0.0681	-0.018	0.017	0.0245	44.2	42,592
	OW_F	0.0001	0.0012	0.030	0.0344	**100**	**42,628**
	OW_X	**0.0001**	**0.0009**	0.030	0.0344	**100**	42,449
30%	Crude	0.5014	-0.1705	0.017	0.1715	0	**42,628**
	$FS_{F\text{-}equ}$	0.0463	-0.0179	0.010	**0.0242**	56.8	42,597
	$FS_{X\text{-}equ}$	0.0453	-0.0183	0.010	0.0244	56.8	42,459
	$FS_{F\text{-}unequ}$	0.0655	-0.0179	0.017	0.0239	42.9	42,597
	OW_F	0.0001	-0.0012	0.026	0.0305	**100**	**42,628**
	OW_X	**0**	**-0.0015**	0.026	0.0306	**100**	42,488
40%	Crude	0.4955	-0.1678	0.017	0.1687	0	**42,628**
	$FS_{F\text{-}equ}$	0.0466	-0.0141	0.010	**0.0212**	74.2	42,597
	$FS_{X\text{-}equ}$	0.0459	-0.0146	0.010	0.0215	73.6	42,476

	$FS_{F\text{-}unequ}$	0.0659	-0.0141	0.014	0.02	45.3	42,597
	OW_F	0	0.0011	0.024	0.0281	**100**	**42,628**
	OW_X	**0**	**0.0008**	0.024	0.0282	**100**	42,506
50%	Crude	0.4913	-0.1683	0.017	0.1691	0	**42,628**
	$FS_{F\text{-}equ}$	0.0492	-0.0134	0.010	**0.0204**	77.4	42,594
	$FS_{X\text{-}equ}$	0.0487	-0.0138	0.010	0.0206	76.2	42,484
	$FS_{F\text{-}unequ}$	0.0696	-0.0134	0.014	0.0188	45.8	42,594
	OW_F	0	0.0003	0.024	0.027	**100**	**42,628**
	OW_X	**0**	**0.0001**	0.024	0.027	**100**	42,517

* The simulations were conducted earliest before the other simulations. At that time, we did not consider $FS_{X\text{-}unequ}$.

a It is bias, not relative bias, because the true effect was set to be zero

b As true effect was zero, therefore, coverage1 and coverage2 were identical.

c SE were estimated by the delta method via GLMs, the same as Ripollone et al (2020, *Am J Epidemiol*).

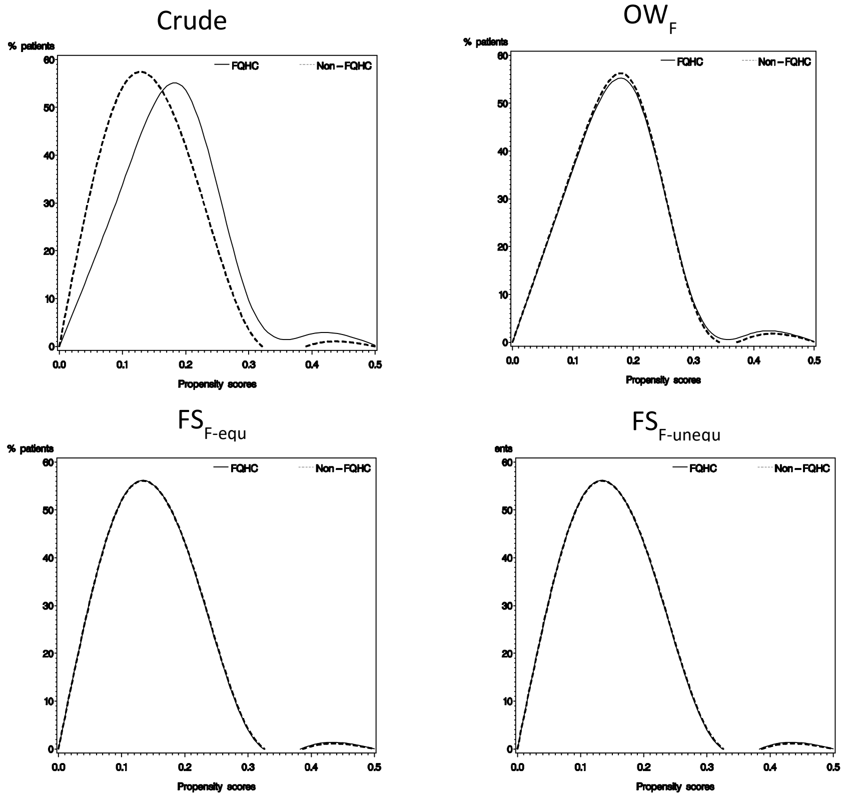

eFigure 1. Plots of distributions of PS per group, associated with four methods: 1) upper left: crude; 2) upper right: $FS_{F\text{-}unequ}$; 3) lower left: $FS_{F\text{-}equ}$; 4) lower right: OW_F.

Footnotes:

1. 'F' = a full set of data
2. 'equ' = ATE with the equal weighting between groups; 'unequ' = ATE with the unequal weighting, where total weight in one group equivalent to the sample size in that group

Finding:

eFigure 1 shows the plots of distributions of PS per group by the crude, OW_F, $FS_{F\text{-}unequ}$, and $FS_{F\text{-}equ}$ methods. The PS distributions of FQHC and non-FQHC groups are fairly overlapped as shown in the crude data. After balancing covariates, the PS distributions are overlapped perfectly in the FS methods and nearly perfectly in the OW method. We did not provide the plots of FS and OW using reduced data because FS_X is very similar to FS_F and OW_X is very similar to OW_F in terms of all the criteria used in the empirical example.

Appendix A. SAS code for analysis of one simulated dataset

A.1 SAS code to obtain OW weights.

```
%macro OW(in_data=, exposure=, ps_cont_var_list=, ps_class_var_list=,
ps_var= , out_data= );
proc logistic data=&in_data desc ;
class &ps_class_var_list;
model &exposure= &ps_cont_var_list &ps_class_var_list;
output out=sample_ps p=&ps_var;
run;

data &out_data; set sample_ps;
      if &exposure = 1 then Overlapwt = 1-&ps_var;
      if &exposure = 0 then Overlapwt = &ps_var;
run;


title 'checking the weights whether they are equal between groups';
proc means data=&out_data sum;
      class &exposure;
      var Overlapwt;
      run;

title;
%mend;
```

A2. SAS code to obtain FS for Average Treatment Effect of the Treated (ATT).

SAS code can be downloaded via the link below.
https://www.drugepi.org/dope/software#Propensity

A3. SAS code to obtain FS-equ weights for ATE.

The main sas code is the same as the code in A2. The only difference is as below.

```
data  strata_n; set  strata_n;
* based on psmatch p49 = p7857 for ATE weighting;
wt_0 = (total_exp + total_unexp) / total_unexp; * where total_exp =
N_total_exp_in_stratum_i, total_unexp = N_total_unexp_in_stratum_i  ;
wt_1 = (total_exp + total_unexp) / total_exp;
run;
```

A4. SAS code to obtain FS-unequ weights for ATE.

The main sas code is the same as the code in A2. The only difference is as below.

```
data  strata_n; set  strata_n;
* Based on Desai and Frankln (2019);
wt_0 = ((total_exp + total_unexp)/(&sum_exp +
&sum_unexp))/(total_unexp/&sum_unexp); * where total_exp = N exposed in
statum i , total_unexp = N unexp in statum i;
wt_1 = ((total_exp + total_unexp)/(&sum_exp +
&sum_unexp))/(total_exp/&sum_exp);
run;
```

A5. SAS code to obtain Mahalanobis balance.

```
%macro MB(indata=, weight=, var_list=);
proc corr data=&indata cov out=cov_wt (where=(_type_='COV') drop = _name_);
      var &var_list;
      weight &weight;
      run;
proc means data=&indata;
      class exposure;
      var &var_list;
      weight &weight;
      output out=mean_wt (where =(_TYPE_=1)) mean=;
      run;

data mean_wt; set mean_wt; keep &var_list; run;
data cov_wt; set cov_wt; keep &var_list; run;

proc iml;

      use cov_wt;
      read all var _ALL_ into cov;

      use mean_wt;
      read all var _ALL_ into X;

      x0 = X[1,]; *row vector for control group;
      x1 = X[2,]; *row vector for intervention group;

      start mahal1(x1, x0, cov);
         y = (x1 - x0)`; /* col vector */
         d2 = y` * ginv(cov) * y; /* explicit inverse. Not optimal */
         return (sqrt(d2));
      finish;

      md = mahal1(x1, x0, cov);
      print md;

      wt ="&weight";
      n_sim="&i    ";
      create maha var {wt md n_sim};
      append ;
      close maha;
run;

proc append base=out.maha data= maha force; run;
      dm 'log;clear;output;clear;';
%mend;
```

A6. SAS code to model outcome by weighted GLM with cluster-robust standard error method to estimate SE of the effect.

```
proc genmod data=modeltemp desc;
      class id exposure/param=ref ref=first;
      model &outcome= exposure/dist=poisson link=log;
      weight Overlapwt;
      repeated subject = id /type=unstr;
```

```
ods output GEEEmpPEst =Ow_beta NObs = OW_n;
run;
```

Appendix B. Implementation Steps of Plasmode Simulation and the R function

Based on the R Package 'Plasmode' from Authors: Franklin JM, Abdia Y, Wang SV (downloaded via the link: https://cran.r-project.org/src/contrib/Archive/Plasmode/) (the actual R function also copied/pasted below)

First, conduct a logistic regression model using the original data to estimate the associations between the observed covariates and the observed outcome.

Second, with the true exposed effect, obtain new coefficients of covariates by the logistic regression model.

Third, with the true event rate, get approximate desired event rate under the new coefficients.

Fourth, simulate outcome based on the approximate desired event rate in the third step.

# R function used in the simulation study

```
PlasmodeBin_ww<- function(formulaOut=NULL,
objectOut=NULL,formulaExp=NULL,objectExp=NULL,data, idVar,
                          effectOR =1, MMOut=1,MMExp=1, nsim, size,
eventRate=NULL, exposedPrev=NULL)
{
  outcome<- all.vars(form1)[1] ## selects the outcome variable
  exposure<- all.vars(form2)[1] ##selects the exposure variable

  x <- data[order(data[,exposure]),] # order according to exposure
status, unexposed first
  n <- nrow(x)
  n1 <- sum(x[,exposure])      # number of exposed in real data
  n0 <- n - n1


  size1 <- round(ifelse(is.null(exposedPrev), n1*(size/n),
size*exposedPrev))  # desired number of exposed
  size0 <- size - size1
  #if(size1 > n1 | size0 > n0) stop("Number of requested exposed or
unexposed exceeds observed number -- reduce size")



  # estimate logit model for probability of outcome
  modOutBin <- glm2(formulaOut, family = "binomial",
data=x,control=glm.control(trace=TRUE))
  ## Design Matrix used for outcome logistic regression
  X <- model.matrix(modOutBin)


  # find event rate in base cohort
```

```
  if(is.null(eventRate)) eventRate <- mean(x[,outcome])


  # find intercept value needed to get approximate desired event rate
under new parameters
  bnew <- c(coef(modOutBin)[1], MMOut*coef(modOutBin)[-1])
  bnew <- replace(bnew, names(coef(modOutBin)) == exposure,
log(effectOR))
  Xbnew <- as.vector(X %*% bnew)
  fn <- function(d) mean(1 - 1/(1 + exp(d+Xbnew))) - eventRate
  delta <- uniroot(fn, lower = -20, upper = 20, extendInt =
"yes")$root  # one dimensional root (zero) finding
  ##### https://stackoverflow.com/questions/38961221/uniroot-solution-
in-r
  pnew <- 1 - 1/(1 + exp(delta+Xbnew))  # to get approximate desired
event rate under new parameters
  rm(modOutBin)  # remove function

  ### sample and simulate
  ids <- ynew <-data.frame(matrix(nrow = size, ncol = nsim))
  RR <- RD <- vector('numeric', length = nsim)
  for(sim in 1:nsim) {
    idxs0 <- sample(1:n0, size0, replace = TRUE) # sample unexposed
(located in rows 1:n0 of x)
    ids[1:size0,sim] <- x[idxs0, idVar]
    idxs1 <- sample(n0+1:n1, size1, replace = TRUE) # sample exposed
(located in rows n0 + 1:n1 of x)
    ids[size0+1:size1,sim] <- x[idxs1, idVar]
    ids[size0+1:size1,sim]
    ynew[,sim] <- rbinom(size, 1, pnew[c(idxs0,idxs1)])
    datasim <-X[c(idxs0,idxs1),]
    datasim[,2] <- 1
    p_1 <- plogis(as.vector(datasim %*% bnew + delta))
    datasim[,2] <- 0
    p_0 <- plogis(as.vector(datasim %*% bnew + delta))
    RR[sim] <- mean(p_1)/mean(p_0)
    RD[sim] <- mean(p_1)-mean(p_0)
  }

  ARR <- mean(RR)
  ARD <- mean(RD)
  ## Creating simulated data for the outcome variable
  names(ids) <- paste("ID", 1:nsim, sep = "")
  names(ynew) <- paste("EVENT", 1:nsim, sep = "")
  sim_out_bin <- data.frame(ids, ynew)

  # TrueOutBeta <- bnew
  # RR <- ARR
  # RD <- ARD
  #
  # setting <- c(TrueOutBeta,RR,RD)
```

```
  return(list(TrueOutBeta = bnew, RR=ARR,RD=ARD,Sim_Data =
sim_out_bin))

} # End of Function PlasmodeBin_ww
```

# Call the function for the simulation study

```
form1<- ipvst ~ exposure + age + tot_elg_months + total_mc_month +
elixhauser + distance + tanf + urb + insulin + female + race_0 +
race_1 + race_2 + race_3 + race_4    + elg_2

set.seed(32323)

# to generate the simulations samples with 500 simulations, each with
a sample size of 4000, outcome risk of 30%, exposure prevalence of 1%,
and the true treatment effect size of 1

plasm_results <- PlasmodeBin_ww(formulaOut=form1,
objectOut=NULL,formulaExp=NULL,objectExp=NULL, data=DM, idVar="id",
                               effectOR =exp(1),
MMOut=c(1,1,1,1,1,1,1,1,1,1,  1,1,1,1,1,1),
                               MMExp=1, nsim=500, size=4000,
eventRate=0.3, exposedPrev=0.01)


setting <- c(plasm_results$TrueOutBeta, plasm_results$RR,
plasm_results$RD )
```

Appendix C. Evaluation of Number of Simulations

To evaluate whether the number of simulations (i.e., iterations) is enough, we used the formula of number of iterations (denoted by N) needed for Monte Carlo simulations as follows:

$$N = [Z_{\alpha/2}S/E],$$

where $Z_{\alpha/2}$ is the critical value of the normal distribution at $\alpha/2$, α is type I error of 5%, S is Monte Carlo errors (MCE) in the setting, and E is the desired margin of error.[4] As E increases, the number of iterations decreased.

To be conservative, we chose the smallest difference between OW and FS as our margin of error (E) in Tables 2 and 3 respectively. Also, for the choice of S, we did two ways: one was based on mean of all MCE in each eTable and the other was based on its maximum. These calculations and choices of S and E were all included at the bottom of eTables 5 and 6.

REFERENCES:

1. Myers JA, Louis TA. Comparing treatments via the propensity score: stratification or modeling? *Health Serv Outcomes Res Methodol.* Mar 2012;12(1):29-43.
2. Abdia Y, Kulasekera KB, Datta S, Boakye M, Kong M. Propensity scores based methods for estimating average treatment effect and average treatment effect among treated: A comparative study. *Biom J.* Sep 2017;59(5):967-985.
3. Desai RJ, Franklin JM. Alternative approaches for confounding adjustment in observational studies using weighting based on the propensity score: a primer for practitioners. *BMJ.* Oct 23 2019;367:l5657.
4. Oberle W. Monte Carlo Simulations: Number of Iterations and Accuracy. *US Army Research Laboratory.* 2015;https://apps.dtic.mil/sti/pdfs/ADA621501.pdf.
